# Increased risk of atherosclerosis associated with pesticide exposure in rural areas in Korea

**DOI:** 10.1371/journal.pone.0232531

**Published:** 2020-05-01

**Authors:** Sungjin Park, Jung Ran Choi, Sung-Kyung Kim, Solam Lee, Kyungsuk Lee, Jang-Young Kim, Sung-Soo Oh, Sang-Baek Koh

**Affiliations:** 1 Department of Occupational and Environmental Medicine, Cheonan Medical Center, Cheonan, Korea; 2 Institute of Genomic Cohort, Wonju College of Medicine, Yonsei University, Wonju, Korea; 3 Department of Occupational and Environmental Medicine, Wonju Severance Christian Hospital, Wonju College of Medicine, Yonsei University, Wonju, Korea; 4 Department of Preventive Medicine, Wonju College of Medicine, Yonsei University, Wonju, Korea; 5 Institute of Occupational and Environmental Medicine, Wonju College of Medicine, Yonsei University, Wonju, Korea; 6 National Academy of Agricultural Science, Rural Development Administration, Jeonju, Korea; 7 Division of Cardiology, Internal Medicine, Wonju College of Medicine, Yonsei University, Wonju, Korea; 8 Center for Global Health and Social Medicine, Institute of Poverty Alleviation and International Development, Yonsei University, Wonju, Korea; IPATIMUP/i3S, PORTUGAL

## Abstract

Atherosclerosis is a progressive inflammation in systemic vessels, and pesticide exposure has been emerging as its risk factor. This cross-sectional study investigated the association between pesticide exposure and the risk of atherosclerosis in a rural population in Korea using carotid intima-media thickness (CIMT). This study used dataset from the baseline survey of the Korea Farmers Cohort Study between November 2005 and January 2008, and the final analysis included 477 participants. Well-structured questionnaires were used to estimate pesticide exposure. CIMT ≥ 0.9 mm was established for carotid atherosclerosis. Multiple logistic regression analyses were undertaken to evaluate the association between pesticide exposure and atherosclerosis, adjusting demographic and health-related confounders. Even after adjustments, the increased risk of atherosclerosis was significantly associated with pesticide exposure, such as a lifetime history of farming (odds ratio [OR] 3.25 95% confidence interval [CI] 1.51–6.98), a history of using pesticide (OR 3.42 95% CI 1.63–7.16), using pesticide 10 times or more annually (OR 2.55 95% CI 1.21–5.39), and higher cumulative exposure index level (OR 3.63 95% CI 1.65–7.97). Further prospective studies are required to elucidate effects of pesticide exposure on the risk of atherosclerosis.

## Introduction

Atherosclerosis is a systemic disorder that develops gradually and is characterized by chronic inflammation of the blood vessels [1], which may lead to fatal complications such as myocardial infarction or coronary artery disease [2]. Several risk factors for atherosclerosis include family history, aging, obesity, physical inactivity, hypertension, diabetes mellitus, and hyperlipidemia [3].

With the recently growing concern and interest for environmental chemicals, pesticide exposure is emerging as a risk factor for atherosclerosis [4,5]. Although pesticide exposure has been linked with cardiovascular diseases [6,7] and serum levels of organochlorine (OC) pesticides are associated with several forms of atherosclerosis [8,9], the association between pesticide exposure and atherosclerosis is unclear. Moreover, previous studies on this issue have not provided consistent results [10], prompting the need for further investigation.

One main reason for the insufficient evidence in this field would be adversities to accurately assess exposure to pesticides. The exposure assessment using biomarkers of pesticides has been used restrictively due to the low specificity of metabolites and technological constraints [11]. As an alternative option, the assessment of pesticide exposure using questionnaires of patient recall of exposure is the only available approach to estimate cumulative exposure in lifetime [12]. This alternative measurement of pesticide exposure has been widely utilized to evaluate the effects of pesticides on several health outcomes [13,14].

Therefore, this study aimed to explore the association between pesticide exposure and the risk of atherosclerosis using a questionnaire which has been validated to measure exposure to pesticides [15,16]. Since carotid intima-media thickness (CIMT) is a useful indicator of atherosclerosis [17] and cardiovascular events [18], the risk of atherosclerosis was estimated using CIMT.

## Methods and materials

### Study population

This study is a part of the Korean Genome and Epidemiology Study (KoGES), a population-based prospective study aimed to identify risk factors of widespread chronic diseases such as hypertension, diabetes, metabolic syndrome, cardiovascular disease, and atherosclerosis. KoGES has been employed for a wide range of research on health problems of rural populations [19,20]. This cross-sectional study was conducted based on baseline survey data of the Korea Farmers Cohort Study. The first wave was conducted between November 2005 and January 2008.

**Fig 1:**
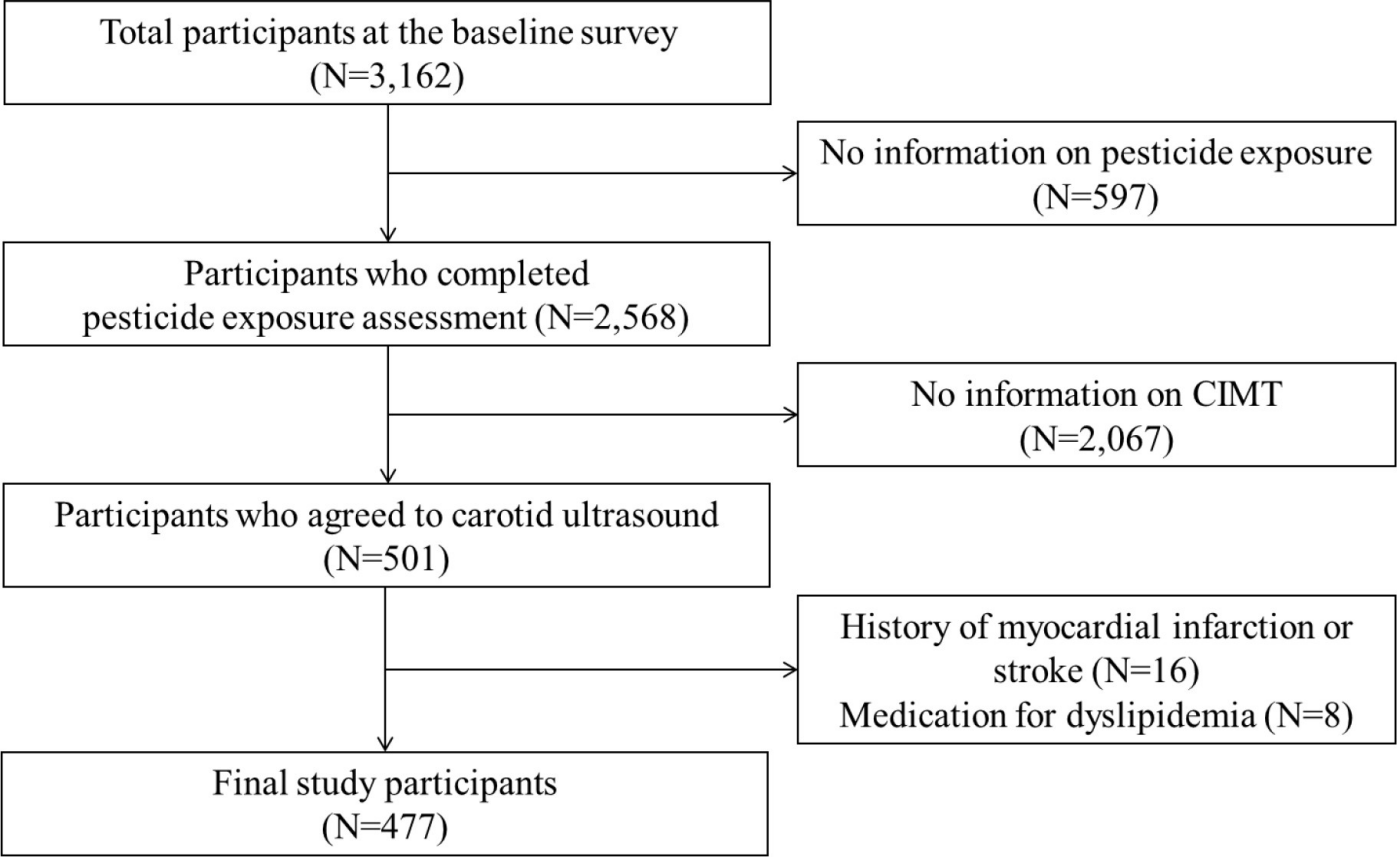
Flowchart of study population the KoGES data.

Participants of the study were the residents of the rural regions of Wonju and Pyeongchang, Gangwon-do, Korea, comprising farmers, farm managers, and other occupations. All participants provided written informed consent prior to enrollment. Of the total 3,162 participants who were included in the baseline survey, 2,568 remained after excluding those with no available information on pesticide exposure. Schedule unavailability and space limitations in the carotid ultrasonography clinic decreased the number of participants to 501 individuals, each of whom voluntarily agreed to undergo carotid ultrasonography. Finally, 477 individuals were included in the statistical analysis after 16 participants with a history of myocardial infarction or stroke and 8 who were on medication for dyslipidemia were excluded (Fig 1). This study was approved by the Institutional Review Board of Wonju Severance Christian Hospital.

### Data collection

Participants of the baseline survey were interviewed by skilled interviewers using standardized questionnaires and inspected by thorough medical examinations. The questionnaires included age, sex, personal medical history (cardiovascular events such as myocardial infarction and stroke and pharmacological treatment of hypertension, diabetes, and dyslipidemia), and lifestyle behavior (smoking, drinking, and exercise). Comprehensive health examinations included anthropometric and blood pressure measurement, venous blood sampling, urine analysis, and carotid ultrasonography for CIMT. Additional details about data compilation have been mentioned elsewhere [21]. Information on pesticide exposure was collected using a revised questionnaire developed by the Agricultural Health Study [22].

### Covariates

Participants were divided into three groups based on their ages: 1) 39–49 years, 2) 50–59 years, and 3) ≥60 years. Obesity was defined as body mass index of ≥25 kg/m^2^. Current smoking, alcohol consumption, and exercise were treated as dichotomous variables. Current medication for chronic diseases, such as hypertension and diabetes, was also included in the analysis.

### Assessment of pesticide exposure

Interviewers used a modified questionnaire developed by the Agricultural Health Study [22] to obtain information about pesticide use or exposure from the respondents. Participants were asked about their experience in farming, using pesticides, as well as the number of years of pesticide use and the number of days of pesticide use in a year.

Because multiple factors may influence the level of exposure to pesticides, the questionnaires for pesticide exposure assessment include action of pesticide use (e.g., application, mixing), mode of application (e.g., hand spray, backpack), use of personal protective equipment (PPE) (e.g., rubber gloves, respirators), and personal work practices and hygiene (e.g., changing into clean clothes or taking a bath after pesticide use). Accordingly, the intensity of exposure and cumulative exposure index (CEI) were generated as follows [22]:

Intensitylevel=(mixingstatus+applicationmethod+repairstatus)×PPE

CEI=intensitylevel×duration(numberofyears)×frequency(averagedaysperyear)

For mixing status, two groups were made (never mixed and mixed, rated 0 and 9, respectively), and eight categories were grouped for the application method (never applied, aerial-aircraft, in furrow/banded, boom on tractor, backpack, hand spray, mist blower/fogger, and airblast, which had six scales, assigned 0, 1, 2, 3, 8, 9, 9, and 9, respectively). Repair status of equipment was divided into two groups (yes or no), and PPE was scored according to the type and use of protective equipment. Supplemental information has been provided elsewhere [22,23].

Several factors related with pesticide exposure were included in the analysis, such as one’s experience of being a farmer, of using pesticides, the number of years and days of using pesticide, and CEI of pesticide exposure. Both the duration (<1, 1–20, and ≥20 years) and frequency (0, 1–10, and ≥10 average days per year) of pesticide use were divided into three groups each. Similarly, both intensity level and CEI of pesticide exposure were split into three groups using the median value (0, lower than the median, and higher than the median).

### Measurement of CIMT & atherosclerosis

As mentioned previously [24], a “brightness” mode ultrasound system (Vivid 7, General Electric Vingmed) and a transducer with 12 MHz frequency and longitudinal two-dimensional ultrasonography imaging were used to inspect the carotid artery. The imaging technique of ultrasound determined the thickness of the bilateral carotid intima-media. The far wall of the carotid artery was separated into two bright lines by a hypoechoic space. A software for semi-automated edge detection was applied to determine CIMT between the leading edge of the first bright line and that of the second bright line (between lumen-intima interface and media-adventitia interface). Values of CIMT exceeding 2 cm were assessed 1 cm proximal to the bulb. The average of maximal values of CIMT on the bilateral sides was used as an indicator of subclinical atherosclerosis [21,25].

Because CIMT is regarded as a predictor of cardiovascular incidents and is often used as a marker of subclinical atherosclerosis [26], we defined atherosclerosis as CIMT ≥0.9 mm [21,27].

### Statistical analysis

SAS software version 9.4 (SAS Institute, Inc., Cary, North Carolina) was applied to all analyses. Chi-square tests were used to show significant associations between CIMT and pesticide exposure. Multiple logistic regression analyses were used to compute odds ratios (ORs), 95% confidence intervals (CIs), and c-statistic. Adjusted models included several risk factors for atherosclerosis, including sex, age, obesity, smoking status, alcohol intake, regular exercise, and pharmacological treatment of hypertension or diabetes. C-statistic is an indicator with predictive power considered equal to that of the receiver operating characteristic curve. It ranges from 0.5 to 1, where a randomly predictive model has a value of 0.5 and a perfectly predictive model has a value of 1. For all analyses, p-value <0.05 was determined as having statistical significance.

## Results

Table 1 describes general characteristics of the study population. Most participants in this study were men; were in their 50s; were of normal weight; and did not smoke, drink alcohol, or exercise regularly. Only 13% of the population was being treated pharmacologically for hypertension or diabetes, and 9% had an increased CIMT of ≥0.9 mm. A comparison of the groups with and without CIMT measurement data is shown in S1 Table. Among all variables, only age group differed significantly between the two groups (p = 0.0431); the individuals with CIMT measurements were younger than those without CIMT measurements. This indicated possible underestimation of CIMT risk due to the generally younger participants of the group with CIMT measurements.

**Table 1:** General description of the study population (N = 477).

Variables	N (%)
Sex	
Men	334 (70.02)
Women	143 (29.98)
Age (years)	
39–49	166 (34.8)
50–59	172 (36.06)
≥60	139 (29.14)
Body mass index (kg/m^2^)	
<25	281 (59.03)
≥25	195 (40.97)
Current smoking	
No	393 (83.26)
Yes	79 (16.74)
Drinking alcohol	
No	255 (53.80)
Yes	219 (46.20)
Current exercise	
No	345 (72.94)
Yes	128 (27.06)
Treatment of hypertension or diabetes	
No	415 (87.0)
Yes	62 (13.0)
CIMT	
<0.9 mm	434 (90.99)
≥0.9 mm	43 (9.01)

Abbreviation: CIMT, carotid intima- media thickness

Table 2 shows factors related to pesticide exposure that were significantly associated with increased CIMT. Number of participants with CIMT of ≥0.9 mm was significantly higher in those who had been a farmer, a history of pesticide use, or higher or lower cumulative exposure.

**Table 2:** CIMT according to pesticide exposure.

Variables	CIMT, N (%)		*p*-value*
	≥0.9 mm	<0.9 mm	
Farmer			0.003
Never	12 (5.04)	226 (94.96)	
Yes	31 (12.97)	208 (87.03)	
Pesticide use			0.002
Never	16 (5.63)	268 (94.37)	
Yes	27 (13.99)	166 (86.01)	
Years of pesticide use			0.210
<1	23 (7.40)	288 (92.60)	
1–20	4 (10.00)	36 (90.00)	
≥20	16 (12.70)	110 (87.30)	
Frequency of pesticide use (per year)			0.056
0	22 (6.98)	293 (93.02)	
1–10	4 (9.09)	40(90.91)	
≥10	17 (14.41)	101 (85.59)	
Intensity level of pesticide exposure			0.133
None	20 (6.92)	269 (93.08)	
Lower exposure	13 (12.87)	88 (87.13)	
Higher exposure	10 (11.49)	77 (88.51)	
CEI of pesticide use			0.006
None	18 (6.19)	273 (93.81)	
Lower cumulative exposure	7 (10.14)	62 (89.86)	
Higher cumulative exposure	15 (17.24)	72 (82.76)	

Abbreviations: CIMT, carotid intima-media thickness; CEI, cumulative exposure index

Intensity level = (mixing status + application method + equipment repair status) × personal protective equipment

Cumulative exposure index = intensity level × spraying years × spraying days per year

*p-value from chi-square test

Table 3 shows ORs and 95% CIs for atherosclerosis according to pesticide exposure from the model adjusted for sex, age, obesity, smoking, alcohol intake, exercise, and the pharmacological treatment of hypertension or diabetes. In the adjusted model, those who had been a farmer (OR 3.25 95% CI 1.51–6.98), used any pesticides (OR 3.42 95% CI 1.63–7.16), used pesticides for 10 times a year or more (OR 2.55 95% CI 1.21–5.39), and had a higher CEI (OR 3.63 95% CI 1.65–7.97) were significantly associated with an increased risk of atherosclerosis. The risk of atherosclerosis was not significantly associated with the prolonged duration of pesticide use, pesticide use of <10 times per year, and intensity level of pesticide exposure.

**Table 3:** Association between increased risk of atherosclerosis and pesticide exposure.

Variables	Crude OR (95% CI)	*p*-value	c-statistics	Adjusted OR (95% CI)*	*p*-value	c-statistics
Farmer			0.621			0.712
Never	1.00 (reference)			1.00 (reference)		
Yes	**2.81 (1.40**–**5.61)**	**0.004**		**3.25 (1.51**–**6.98)**	**0.003**	
Pesticide use			0.623			0.714
Never	1.00 (reference)			1.00 (reference)		
Yes	**2.72 (1.43**–**5.21)**	**0.002**		**3.42 (1.63**–**7.16)**	**0.001**	
Years of pesticide use			0.568			0.675
<1	1.00 (reference)			1.00 (reference)		
1–20	1.39 (0.46–4.25)	0.562		1.56 (0.48–5.03)	0.458	
≥20	1.82 (0.93–3.58)	0.082		2.00 (0.95–4.19)	0.067	
Frequency of pesticide use (per year)			0.589			0.690
0	1.00 (reference)			1.00 (reference)		
1–10	1.33 (0.44–4.06)	0.615		1.54 (0.48–4.89)	0.467	
≥10	**2.24 (1.15**–**4.39)**	**0.019**		**2.55 (1.21**–**5.39)**	**0.014**	
Intensity level of pesticide exposure			0.581			0.682
None	1.00 (reference)			1.00 (reference)		
Lower exposure	1.99 (0.95–4.16)	0.069		2.13 (0.95–4.79)	0.067	
Higher exposure	1.78 (0.79–3.89)	0.172		2.14 (0.89–5.14)	0.089	
CEI of pesticide use			0.613			0.713
None	1.00 (reference)			1.00 (reference)		
Lower cumulative exposure	1.61 (0.66–3.96)	0.297		1.94 (0.75–5.02)	0.174	
Higher cumulative exposure	**2.98 (1.46**–**6.06)**	**0.003**		**3.63 (1.65**–**7.97)**	**0.001**	

Abbreviations: CEI, cumulative exposure index; OR, odds ratio; CI, confidence interval

Intensity level = (mixing status + application method + equipment repair status) × personal protective equipment

Cumulative exposure index = intensity level × spraying years × spraying days per year

*Adjusted for sex, age, obesity, smoking, alcohol intake, exercise, pharmacological treatment of hypertension or diabetes

**Bold** text shows the statistical significance of the odds ratios

## Discussion

This study’s findings suggest that the increased risk of atherosclerosis was significantly associated with pesticide exposure using CIMT and pesticide exposure assessment based on questionnaires. The pesticide exposure-related factors, including farming history, history of pesticide use, pesticide use of ≥10 times per year, and a higher level of CEI, were significantly associated with increased CIMT, after adjustment for sex, age, obesity, smoking status, alcohol intake, regular exercise, and current medication for hypertension or diabetes.

The result of this study seems similar to that of recent studies. A previous study reported that OC pesticides were related to the higher prevalence of peripheral artery diseases [8] and that elevated serum levels of OC pesticides were associated with the risk of atherosclerosis [9]. Meanwhile, other researchers found contrasting results wherein lower mortality from coronary artery disease was seen with higher exposure to herbicides [10]; and no significant relationship was reported between OC pesticides, polychlorinated biphenyls, and CIMT [4].

Atherosclerosis is accompanied by the accumulation and oxidation of low-density lipoproteins (LDLs) in the vessel walls. The inflammatory process begins at the site of oxidation with the accumulation of pro-inflammatory cytokines and immunocytes such as monocytes and T-lymphocytes [4].

Although the mechanism between pesticide exposure and atherosclerosis development is barely understood, increased levels of oxidative stress and alterations in lipid homeostasis may explain this process. Some experimental evidence suggests that OC and organophosphate (OP) pesticides contribute to the development of atherosclerosis through increased levels of reactive oxygen species, depletion of antioxidant defensive system, and increased peroxidation of lipids [28]. The findings of one study of Korean farmers [21] were in accordance with the abovementioned mechanism, which elucidated the role of oxidative stress in the pathway from pesticide exposure to subclinical atherosclerosis. Polychlorinated biphenyls pesticide was observed to influence the development of atherosclerosis by mediating endothelial dysfunction and increasing sensitivity to oxidative stress [8]. Additional evidence from experimental data suggested that OC pesticides accelerate the atherosclerotic process via direct oxygen toxicity to oxidize LDLs into a pro-atherogenic form and that OP pesticides disrupt the uptake and transport of cholesterols [28].

In this study, CIMT of ≥0.9 mm was employed to define atherosclerosis as it was validated by several studies [21,26,29]. CIMT is used as an indicator of progressive changes of atherosclerosis in general vessels as well as coronary arteries [26,30]. For patients with medical disorders, increased CIMT can be used as a complementary predictor of cardiovascular events [18,31]. Although it has been widely used because of its simplicity and usefulness, it also has several drawbacks, including the lack of standardized approach for measurement and low accuracy in assessing atherosclerosis. Therefore, future studies including other complementing markers such as carotid plaque and artery intima-media complex as well as CIMT are necessary to reflect diverse aspects of atherosclerosis.

Our study has several limitations. First, the nature of this study contained a cross-sectional design and thus could not establish a causal relationship; however, our findings can show the association between atherosclerosis and pesticide exposure, prompting the need for research on other possible risk factors. Second, detailed information on pesticides including their types, half-lives, and biological mechanisms was not included. At the survey phase of collecting information, participants were asked to answer specific questions on the duration and the types of pesticides they used, including the names of chemicals or products, partial product names, manufacturer names, targets or purposes, and formulation of products. Unfortunately, they had difficulty in describing them accurately [32] and, thus, most of them failed to answer the items correctly. However, studies have been performed on health effects of pesticide exposure using this method to evaluate exposure without the information on the type of pesticides [13,14,33,34]. Finally, only 501 of 2568 (approximately 20%) individuals who completed the pesticide exposure assessment agreed to undergo CIMT measurements for inclusion in this study. However, as shown in S1 Table, the general characteristics (except for age group) and pesticide-related factors did not differ significantly between the study population and the excluded group without CIMT measurement. Therefore, the selection bias in this study is considered not substantial, although it cannot be neglected.

This study suggests that pesticide exposure, which may occur occupationally in agricultural settings, plays a role in the development of atherosclerosis. Prospective studies with a larger sample size should be conducted to further investigate the effects of pesticide exposure on the risk of atherosclerosis.

## Supporting information

S1 Dataset (XLS)

S1 File (DOCX)

S2 File (DOCX)

S1 Table General characteristics of the participants who underwent CIMT measurements (study population) versus those who did not undergo CIMT measurements (excluded population). (DOCX)
