# Supplementary material for: Increased risk of atherosclerosis associated with pesticide exposure in rural areas in Korea
Source: PLoS One. 2020 May 1;15(5):e0232531. doi: 10.1371/journal.pone.0232531 (PMC7194402; doi:10.1371/journal.pone.0232531)
Supplement: S1 Table — (DOCX) [file pone.0232531.s004.docx]

| **Supplementary Table 1.** **General characteristics of the participants who underwent CIMT measurements (study population) versus those who did not undergo CIMT measurements (excluded population)** | | | |
| --- | --- | --- | --- |
| Variables | CIMT measurement [N (%)] | | p-value |
|  | Without (N=1984) | With (N=477) |  |
| Sex |  |  | 0.2289 |
| Men | 797 (40.17) | 206 (43.19) |  |
| Women | 1187 (59.83) | 271 (56.81) |  |
| Age (years) |  |  | 0.0431 |
| 39–49 | 579 (29.18) | 165 (34.59) |  |
| 50–59 | 735 (37.05) | 173 (36.27) |  |
| ≥60 | 670 (33.77) | 139 (29.14) |  |
| Body mass index (kg/m^2^) |  |  | 0.7837 |
| <25 | 1179 (59.64) | 280 (58.95) |  |
| ≥25 | 798 (40.36) | 195 (41.05) |  |
| Current smoking |  |  | 0.3763 |
| No | 1681 (84.9) | 393 (83.26) |  |
| Yes | 299 (15.1) | 79 (16.74) |  |
| Drinking alcohol |  |  | 0.1350 |
| No | 1139 (57.58) | 255 (53.8) |  |
| Yes | 839 (42.42) | 219 (46.2) |  |
| Marital status |  |  | 0.2411 |
| Married | 1745 (88.35) | 426 (90.25) |  |
| Others | 230 (11.65) | 46 (9.75) |  |
| Educational level |  |  | 0.5709 |
| Below middle school | 957 (48.5) | 224 (47.06) |  |
| Middle school or above | 1016 (51.5) | 252 (52.94) |  |
| Income level |  |  | 0.9790 |
| <1,500,000 Korean Won | 1016 (57.6) | 252 (57.67) |  |
| ≥1,500,000 Korean Won | 748 (42.4) | 185 (42.33) |  |
| Current exercise |  |  | 0.4472 |
| No | 1472 (74.16) | 344 (72.88) |  |
| Yes | 513 (25.84) | 128 (27.12) |  |
| Treatment of chronic diseases |  |  | 0.9041 |
| No | 1722 (86.79) | 415 (87.00) |  |
| Yes | 262 (13.21) | 62 (13.00) |  |
| Farmer |  |  | 0.1004 |
| Never | 907 (45.72) | 238 (49.9) |  |
| Yes | 1077 (54.28) | 239 (50.1) |  |
| Pesticide use |  |  | 0.5751 |
| Never | 1049 (58.12) | 284 (59.54) |  |
| Yes | 756 (41.88) | 193 (40.46) |  |
| Years of pesticide use |  |  | 0.1866 |
| <1 | 1258 (63.41) | 312 (65.41) |  |
| 1–20 | 219 (11.04) | 39 (8.18) |  |
| ≥20 | 507 (25.55) | 126 (26.42) |  |
| Frequency of pesticide use (per year) |  |  | 0.6691 |
| 0 | 1290 (65.02) | 316 (66.25) |  |
| 1–10 | 206 (10.38) | 43 (9.01) |  |
| ≥10 | 488 (24.6) | 118 (24.74) |  |
| Intensity level of pesticide exposure |  |  | 0.3822 |
| None | 1224 (61.69) | 289 (60.59) |  |
| Lower group | 368 (18.55) | 101 (21.17) |  |
| Higher group | 392 (19.76) | 87 (18.24) |  |
| CEI of pesticide use |  |  | 0.2283 |
| None | 1224 (65.95) | 291 (65.25) |  |
| Lower group | 325 (17.51) | 68 (15.25) |  |
| Higher group | 307 (16.54) | 87 (19.51) |  |

CEI, cumulative exposure index; CIMT, carotid intima-media thickness
